# The co-production of a workplace health promotion program: expected benefits, contested boundaries

**DOI:** 10.1057/s41285-022-00186-4

**Published:** 2022-08-17

**Authors:** Paolo Rossi, Francesco Miele, Enrico Maria Piras

**Affiliations:** 1 Department of Sociology and Social Research, University of Milano Bicocca, Via Bicocca Degli Arcimboldi 8, 20126 Milan, Italy; 2 Department of Political and Social Sciences, University of Trieste, Piazzale Europa 1, 34127 Trieste, Italy; 3 Fondazione Bruno Kessler, Via Sommarive 18, 38123 Trento, Italy

**Keywords:** Workplace health promotion, Boundary objects, Co-production, Well-being

## Abstract

Workplace health promotion (WHP) are often depicted as an opportunity for pursuing a better and broader well-being condition under the assumption that working environments affect the physical, mental, and social well-being of individuals who spend large proportion of waking hours at work. While most empirical studies provided medical evidence to the effectiveness of WHP programs, scholars question the instrumental purposes of these programs founded on the belief that “healthy workers are better workers”. Little is known, for instance, about the design of WHP programs and their acceptance by workers. Our study addresses this gap, analyzing the co-production of a WHP program in an Italian research institute promoted by the healthcare authority, the local government and the national center for prevention and security in the workplaces. To this aim, we adopt the notion of boundary object investigate how different stakeholders reclaim to take part and being involved in this process, re-shaping their goals and their boundaries and why a WHP program or parts of it may be rejected or re-negotiated by its recipients. Our analysis reveals how each stakeholder contributes to re-shape the WHP program which emerges as the modular product of the composition of each matter of concern. Most notably, the strong rooting in a clinical perspective and the original focus on only workers at risk is gradually flanked by initiatives to involve all employees. Moreover, workers draw a line as for the legitimacy of employers’ intervention in the personal sphere of health promotion, embracing interventions addressing diet and physical activity while rejecting measures targeting smoking and alcohol consumption.

## Introduction

A growing number of companies have been developing workplace health promotion (WHP) programs for their employees. Before the outbreak of the Covid-19 pandemic, most of these programs dealt with one or more of the four pillars of primary prevention (nutrition, physical activity, alcohol consumption and smoking) with the aim of improving the overall well-being of workers and preventing chronic diseases. Although the pandemic has shifted the focus of some programs (or interrupted them), it is possible to state that WHP still represents a relevant benefit for the workers who could enjoy it.

WHP programs represent a broad set of initiatives for the improvement of the health status of individuals and communities, stemmed by long-standing campaigns and movements prompted by different international institutions in the Seventies. Most of these programs promote healthy behaviors, outlining the assumptions that are supposed to drive an individual to a condition of well-being.

WHP is an umbrella-concept that outlines different initiatives that organizations adopt and develop for improving the health status of workers (Shain and Kramer, 2004) under the assumption that working environments affect the physical, mental, and social well-being of individuals who spend “large proportion of waking hours at work” (Sargent et al., 2018, p. 1). Working activities are thus one of the most important “determinants of health and health inequalities” (Bambra et al., 2014, p. 113).

The development of WHP programs requires the involvement of different actors (recipients, providers, public institutions and so on) to converge toward shared objectives. While the definition of good health status pertains to factors that include objective and standardized medical criteria as well as subjective perceptions, the development of these programs requires to design actions sufficiently adaptable for people and social groups with different needs, beliefs, and expectations about their health conditions.

Considering the complexity of this issue, we can frame WHP interventions as boundary objects, “objects which both inhabit several intersecting social worlds” (Star and Griesemer, 1989, p. 393). We argue that conceptualizing WHP programs as boundary objects allows to shed light on the processes through which all the actors involved in their development come to an agreement for designing actions for promoting health in a specific social context.

Within this framework, it is possible to argue that starting a collaboration between the actors, rather than hypothesizing and forecasting the effectiveness of WHP programs (e.g., Goetzel and Pronk, 2010; Schröer et al., 2014; Pedersen et al., 2018), is one of the most challenging issues for health promotion initiatives, although this topic is less studied and debated in the literature and it is overshadowed by the studies on the outcome of WHP programs. To fill this gap, we argue that an analysis of how the actual design of a WHP program unfolds and comes into being is needed. Shading light on how different actors come to agree on common objectives allows for understanding what are healthy behaviors to be promoted and setting the conditions for the collaboration between all the actors interested in this process. The first focus is thus the analysis of the design of WHP programs, as if they were boundary objects that provide the “*capacity to negotiate interests and transform knowledge*” (Carlile 2004, p. 559). This topic can be synthesized into this question: how can different actors come to agree on a shared workplace health prevention program?

The second focus deals with the issue of workers’ compliance with the activities proposed by a WHP program. Specifically, we investigate to what extent workers are willing to comply or, put differently, which are “the boundaries of boundary objects”. Assuming, as we made above, that WHP programs stand on boundary objects that prompt the adoption of healthy behaviors, we are interested in studying how far can a WHP program go with respect to personal health conditions, needs and beliefs to understand the boundaries that health promotion programs should not overcome. This a crucial point for the development of WHP programs since the pursuit of an agreement between the relevant actors does not necessarily mean reaching a generalized consensus. The recipients are free to reject or not being with compliant with the program. It is moreover important to remind that the health conditions of recipients are very different, and they can attach different meaning to the activities proposed by WHP programs.

This topic can be synthesized in the following second question: on what terms a workplace health promotion program become acceptable by its main intended beneficiaries, the employees?

To answer these questions, we shall adopt a theoretical framework building on two scholarly traditions. On the one hand, we shall use, as said earlier, the notion of boundary object to follow the trajectory of the WHP program across different settings and its modifications over time. On the other hand, we will frame the process of the making of the WHP program as a co-production endeavor, shading light on the mix between cooperation and conflict among the several actors involved with particular regard to the end-users.

## The notion of boundary object and its fitness for WHP

The notion of boundary object has been introduced by Star and Griesemer (1989). The authors identified four types of boundary objects: repositories, ideal types, coincident boundaries, and standardized forms. As stressed later by Star (2010), boundary objects represent arrangements that should facilitate the cooperation between different groups even without consensus between them. The authors emphasized this latter point: from their point of view, consensus is not a prerequisite for starting a collaboration, neither a condition that necessarily has to be reached at the end of or during a collaboration. Star and Griesemer rather argued that a fruitful collaboration needs consistency across intersecting social worlds (1989) and boundary objects emerge and are worked on to satisfy the informational requirements of each of the social worlds involved in a collaboration (*ibidem*).

The notion of boundary objects has been discussed and developed from several authors who, starting from the work of Star and Griesemer, expanded this concept, proposing further types, definitions, and fields of application (e.g., Carlile, 2004; Kimble et al., 2010; Rehm and Gol, 2015).

The notion of boundary object further sheds light on the relational dimension of the arrangements that support the collaboration between actors who stand at different positions in a social environment and express heterogeneous needs and interests about an issue that crosses institutional, organizational, and professional boundaries, such as WHP programs (McLeroy et al., 1988; Henning et al., 2017). This suggests adopting an ecological perspective in studying the collaborations that deal with WHP programs, to “*bring to the fore the wider relational scene of which the phenomenon is part*” (Nicolini, 2011, p. 604). From this point of view, the design of the arrangements that stand behind (or beside) their enactment can be studied from different levels of observation, bridging global movements to local organizational initiatives, zooming from a panoramic view of macro social trends to a fine-grained analysis of microsites of experimentation, merging the different insights that emerge at each layer (Nicolini, 2009).

This zooming allows to intercept different points of view on WHP. A stream of literature criticized the instrumentality of these initiatives. Some scholars argued that the introduction and development of WHP programs is mainly purposed to improve the performances of companies that instrumentally adopt and develop them. This means that caring for the health of workers provides some benefits for the companies too, reducing absenteeism, decreasing of the number of accidents, and increasing individual productivity (Bertera, 1990; Riedel et al., 2001; Rongen et al., 2013; Lindström, 2016; Poscia et al., 2016). Shortly, this argument is consistent with the belief that a healthy worker is a better worker. However, although some WHP programs are supposed to be instrumentally adopted by companies, this does not mean that workers cannot enjoy some benefits from them. Most of the findings of the empirical studies that analyzed the effects of WHP programs on workers’ health report that they reached their purposes and contributed to improve the health status of their recipients, according to the medical parameters adopted for its measurement (Goetzel and Pronk, 2010; Schröer et al., 2014; Pedersen et al., 2018).

At this point, the emerging issue is understanding whether and how it is possible to find consistency between companies’ goals and workers’ needs.

## The co-production of a boundary object: WHP programs as spaces for collaboration and conflict

The notion of co-production is rooted in a double tradition. It stems from the literature on public administration and management promoted by the influential work of Ostrom (1972) and from the studies on service management theory (Osborne et al., 2016). Co-production has been framed as the involvement of citizens and public service professionals in the provision of public services (Verschuere et al., 2012; Nabatchi et al., 2017) and, as such, it represents an innovative pattern of social innovation (Voorberg et al., 2015).

The scope of this notion has been progressively expanded and it now outlines different kinds of experiences where actors who hold convergent or contrasting stakes over an issue adopt collaborative forms of interaction (Osborne et al., 2016, 2018; Tummers et al., 2016). Osborne and colleagues have proposed a taxonomy (2016), based on the distinction between the nature of the co-production (involuntarily or voluntarily) and the locus of co-production (individual or service system). Sorrentino and colleagues distinguish between individual co-production, that provides individual benefits to a single user or client of a service who participate to its design and production, and collective co-production, when the participation is not limited to clients but involve different categories of actors (2018).

From our point of view, two more insights are particularly pertinent for understanding how a WHP program can be developed through a process of co-production. Nabatchi and colleagues (2017) outline that co-production is a form of collaboration that may refer to the delivery as well as to the design of a plan or arrangement (2017), as in our case. In a similar vein, Vennik and colleagues state that co-production can be defined considering not only its outcome, as they claim that it can refer also to an earlier phase of a co-design which “*involves not only designing the functionality, safety and reliability of the product or service, but also the whole interaction with it and how it feels or is experienced by users*” (2016: 153).

These insights match the points considered earlier when discussing the representation of a WHP program as a boundary object. The development of a WHP program can take the form of a co-production process, as it implies both the definition of a plan or arrangement and the shaping of the ongoing interactions between the actors who are involved in its implementation.

From this point of view, all actors should mobilize to promote a shared representation of this boundary object while adapting it to their own needs and preferences. (Bergman et al., 2017). This can challenge their commitment to the development of the program and the boundaries of their participation.

This tension is being discussed in an emerging body of literature, where co-production processes are intended as arenas where cooperation and conflict coexist (Jaspers and Steen, 2017) to the point of stating that co-production is suited for problems where consensus is low (Scolobig and Gallagher, 2021). Co-production processes face value conflicts. Aschhoff and Vogel (2018)[A3] identify three major value tensions (inclusiveness/accountability tension, flexibility/accountability tension, productivity/diversity tension) and several strategies to cope with such tensions. Jaspers and Steen (2017) identify efficiency, individual freedom, reciprocity, and inclusion as common sources of tension among professionals and citizens in co-production.

On these assumptions, the notion of co-production can be adopted as an analytical device for identifying and analyzing the making of a WHP program. The latter, intended here as a boundary object at the core of co-production processes, is shaped through the collaboration between different individuals and communities that, following the state of art, have heterogeneous or even conflicting goals, interests and ethical positions. The aim of this contribution is to understand how the different actors (*in primis* companies and workers) involved in the co-production of a WHP intervention can collaborate across these boundaries reaching an agreement on a shared and acceptable health promotion program.

## Research setting and methodology

This work analyzes the implementation of a WHP program (namely KeyToHealth) promoted by FBK, a multidisciplinary research center in Italy. FBK has more than 500 workers, employed with research and office roles (HR, management, administration).

Over the years, the research center has developed an occupational welfare system mainly addressing work-life balance, training, education, sports as well as health. In this study, we report the development of a WHP project which started as primary prevention program of Type 2 Diabetes and cardiovascular diseases and developed into a more articulated health promotion initiative.

We focus our attention on the design of this program, observing the different actors that were called (or asked) to participate to this process and their relations and “reactions” to the progressive stages of development of the program. We argue that this collaboration took the un-expected form a co-production process.

### The research

The second and the third author were involved as social researchers in charge of supporting the design of the program since its beginning to develop a research action activity to support it. With this aim different qualitative techniques were adopted. The first source of data is the participant observation of the different phases of design and implementation of the program, starting from the presentation of its first draft to its finalization. The second author, who has been the project manager of the program, kept a research diary of all the meetings he participated to. The diary has been kept following a reflexive approach for which also researchers, with their personal attitudes and cultural backgrounds (i.e., assumptions, values and belief systems), can influence and re-direct the co-production dynamics at stake (see: Chambers, 2012).

The second source of data are five focus groups (see Table 1) for gathering some information about the meanings that the initial target group of workers attached to the program. At the end of the focus groups, workers’ feedbacks have been reported to the subjects involved in the design processes (employers and employees, the local government, trade unions and healthcare institutions) to draft the final version of the project.

**Table 1:** Focus Group participants by sex and role

Focus Group	Participants	Researchers	Office workers
1	5 (3 F; 2M)	0	5
2	4 (2 F; 2M)	1	3
3	8 (6 F; 2M)	2	6
4	7 (3 F: 4M)	3	4
5	7 (3 F: 4M)	5	2
Total	31 (17 F; 14M)	11	20

These data where complemented by an analysis of policy documents regarding WHP and their role in regional and national health prevention plans.

The workers who were used to the design and promotion of well-being programs (e.g., members of Health and Safety unit, trade union representatives, members of the internal recreational club) participated to the first and the second focus group. The participants of the other focus groups were workers who asked to participate after receiving an open invitation sent by e-mail to the whole workforce.

Empirical data were analyzed using template analysis (King 1998), a model for coding the content of textual data. This method implies the initial identification of some themes defined by the researchers in accordance with the most discussed topics in the literature. The researchers code the fragments of text they consider more relevant and can create new templates if pre-existing themes do not cover emerging insights. By this way, a system of interconnected categories for interpreting the phenomenon at stake (i.e., the co-production of a WHP program) emerge and provide meaning to the whole process being studied.

## The (un-expected) co-production of a WHP program: the case of the KeyToHealth project

We shall present our findings describing the process of negotiation of the details of the Workplace Health Promotion program, our boundary object. By following the trajectory from its inception (the preliminary idea) to its end (the final protocol) we shall show how the program was modified as new stakeholders were involved so to inhabit each different social world. We shall introduce our findings presenting the institutional context that framed and created the conditions for the WHP initiative to take place.

### The context

In Italy the safeguard of workers has been traditionally limited to occupational safety. However, the program under scrutiny has been promoted in an evolving and more favorable context, when WHP programs started to be promoted at an institutional level by different actors (national government, regions, healthcare authorities).

Without claiming to be exhaustive, we try to summarize the most significant changes at both national and local levels.

The promulgation of act on health and safety at work (known as Legislative Decree N.81/2008) has been important for the diffusion of WHP for two reasons:

- it promoted a broad conception of health, conceiving it as “*the state of complete physical, mental and social well-being, consisting not only of an absence of sickness or sickness*” (art. 2);
- it claimed that occupational physician, in addition to help employers in the protection of health and psychological integrity of workers, can be involved in the implementation and enhancement of volunteers' health promotion programs, "according to the principles of social responsibility” (art. 25).

Moreover, the National Institute for Insurance against Accidents at Work (INAIL) became a member of the *European Network for Workplace Health Promotion*, an international network aimed at supporting the development of WHP in Europe. INAIL has thus become more interested in promoting and financing projects directed at safeguarding the general well-being of workers.

At local level, it is important to mention the “*2015–2020 Local plan for the Health*”, promulgated by the Local Healthcare Authority (LHA). This policy stressed the need of implementing broader organizational programs addressed to the promotion of healthy lifestyles among workers.

In this scenario, the pressures made by national and local bodies for adopting and/or developing WHP programs became stronger, laying the basis for the wide adoption of these initiatives. As well known in organization studies (Czarniawska, Sevón, 2005, 2011), innovative ideas can travel thanks to the intervention of powerful actors able to promise both material (economic resources) and symbolic (prizes awarded by national and local institutions) incentives. However, it is equally well known that, adopting Latour’s words (1986: 267), “the spread in time or space of anything claims, orders, artifacts, goods is in the hands of people; each of these people may act in many different ways, letting the token drop, or modifying it, or deflecting it, or betraying it, or adding to it, or appropriating it”. So, on the one hand, the dynamics through which actors placed in particular organizational contexts decide to adopt and implement a WHP program are locally defined; on the other hand, these local dynamics contribute to the travel on a global scale of WHP, intended as a specific kind of health promotion.

In the organization considered in our study, the idea of implementing a WHP program stemmed from the opportunity to retool some technologies originally designed to collect citizens’ personal health data to support personal health information management (Piras and Miele, 2015; Piras, et al., 2019) and the prevention of chronic illnesses. The retooling was suggested and promoted by the local Department of Health and Social Policies [1] and the Local Health Authority (LHA)[2], the main stakeholders of the research center to replicate WHP initiatives in the region.

In the next pages, we will explore in detail the co-production dynamics through which WHP took shape, shifting from being a vague idea of safeguarding employers’ well-being to a concrete project, thanks to the involvement of a wide range of actors with heterogeneous goals, interests and ethical positions about health promotion.

### The original design of the program

The program was initially conceived as a project defined in clinical terms with the objective to target workers with a high risk of developing cardiovascular diseases and/or Type 2 diabetes. The first working proposal of the program was designed by three members of the research center (the head of the “eHealth unit”, the second author of this article who acted as project manager, the head of the “Safety unit) and the occupational physician.

Three main initiatives were envisioned:

- the retooling of a digital platform previously tested with overweight children and adolescents for educating the target group with respect to nutrition issues;
- a screening, coordinated by the occupational physician, for assessing the health risks of each worker, through a survey and face-to-face meetings;
- the enrollment of a counselor for supporting workers in lifestyle changes.

The first working proposal of the program satisfied the expectations of both organizations, interested in conducting research on a novel topic, and the occupational physician, interested in developing new competencies to be proposed to other customers. Or, to adopt the vocabulary introduced in the previous section, the working proposal was a boundary object that made possible the cooperation among the parties involved.

The group also decided to strengthen the proposal by inviting other actors to join, each with a different motivation:

- The research center’s HR office, to leverage its experience in designing and managing the internal organizational welfare program (e.g., work-life balance policies, recreational and educational activities for workers’ children, agreements with local sports facilities, ironing services, etc.);
- INAIL's local branch, to reinforce the credibility of the initiative by partnering up with the most relevant institution in the promotion of WHP at the national level;
- the Department of Health and Social Policies of the local government and the Local Healthcare Authority (LHA), to involve the research center’s most relevant stakeholders in the crafting of WHP initiatives to be replicated in the region.

The new actors involved agreed on the first working proposal. At this stage the main contribution of the new partners was the cooperation in detailing and revising the questionnaire adopted to select participants to the WHP initiative and, most notably, the request of the LHA to coordinate the activities dealing with the issue of promoting changes in workers’ lifestyles. It was decided that a counselor of the LHA would organize face-to-face meetings with workers to promote healthier lifestyle habits setting personalized nutrition and physical activity targets.

Such specifications to the original proposal were the first initiatives for the co-production of the program. The boundary object was reconfigured to make it capable of mediating across the social worlds of the new actors involved. The co-production involved external agencies that provided legitimacy and accreditation to the program. Internally, the HR office provided stability and consistency to the program with respect to former initiatives. This strategy aimed at stabilizing the program, conforming it to a wider logic of delivery of occupational welfare services.

While the “original proposal with modifications” served well the purpose of allowing the cooperation of all parties involved, an un-expected event led some radical changes.

### The revision of the original proposal: from selection to inclusion

The questionnaire to determine the eligibility to be enrolled in the WHP program was made available electronically to all workers of the research center who could voluntarily take part in the survey. The questionnaire, through a list of standardized items, was intended to identify individuals at high risk of developing type 2 diabetes and cardiovascular diseases. However, only 30 workers (among approximately 500) met the criteria to be included in the program.

Such a result was not surprising for the occupational physician, and it was regarded with favor by the research unit involved, which was used to have pilot experimentations with similar numbers. On the contrary, the other parties considered these low figures not compatible with their involvement. Both the Local Healthcare Authority and the local government believed that the technology could have helped to create affordable workplace health promotion initiatives and make them available for a larger percentage of the workforce. Similar concerns with different motivations were expressed by the HR department. The organizational welfare policy of the research center was to offer services to all the workers, and the HR department pushed to have a more inclusive prevention program, one that could address the needs of the whole workforce. These considerations led to complement the original proposal with additional activities addressed to the whole workforce. In detail, it was decided to host seminars about healthy lifestyles inviting prestigious speakers, make some interventions in the canteen, and add healthy options in the vending machines in the relax areas. The co-production, in this phase, through these additions to the original proposal extended the scope of the WHP initiative from health prevention to well-being promotion and such modification allowed to keep the alignment of the actors around the initiative.

At the same time, the shift from a ‘strictly medical’ intervention to a more lifestyle-oriented program paved the way to include the recipients of the program as a new actor of the process.

This choice has been prompted by the second and the third author, who had already been involved in projects adopting participatory techniques. The assumption that including the potential end-users of the program in its design would pave the way for its success led the researchers to actively involve the workers in its implementation. This belief is well-grounded in the strand of studies concerning co-production that informed the participation of the second and the third author in the project.

### The focus groups with the workers: from passive recipients to vocal actors

At this stage, there was a two-headed program. On the one hand, a preventive initiative framed in clinical terms targeting 30 workers while, on the other hand, a well-being program to be offered to all 500 employees. The first was already defined in detail while the latter was still to be finalized. Five focus groups were thus organized, with a twofold purpose of involving workers in the design of the program and promoting it among the workers.

Each focus group discussed the main goals of the program and all of the activities that would be proposed to workers. Two focus groups were carried out with employees previously involved in the development of other well-being programs. These workers were identified as key informants (i.e., people with first-hand knowledge about the development of well-being services), as well as potential recipients of the new program. The other three focus groups were conducted with employees recruited through an open invitation.

The information gathered from the focus groups outlined mixed reactions to the program. Most workers appreciated the focalization of the program on lifestyles. Many of them reported to have already undertaken different attempts to modify their daily routines, adopting healthier behaviors habits (e.g., sport, diet).

*Interviewer: in the future, if you should enhance the initiatives for the physical activities promoted by the recreational club, what would you do?*

*Worker 1: I think (…) that a comfortable locker room where you can go and get changed after a run would already be a great success. In addition, having the possibility of having a gym with 3–4-5–6-7 machines would be very, very important in my opinion, for letting off the stress…*

*Worker 2: I fully agree. I love biking, and sometimes I go to work by bike (…).*

*Worker 3: I would like interventions promoting the usage of public transport or the moving walk. When I go to work on foot, I risk getting hit … Transforming the lanes around our workplace into pedestrian areas could be a good idea …*

*(Focus group 2 with the workers).*

*Worker 1: in this project, I would start to change the approach. There is a growing desire on the part of the colleagues for a broader involvement in decision making (…). Better communication and greater transparency are needed.*

*(Focus group 1 with the workers).*

However, the focus groups pointed out some discrepancies between the needs and expectations of the workers and the benefits that the program should provide. The workers suggested several solutions to promote healthy lifestyles in the workplace, such as an internal gym for physical activity, and the construction of a pedestrian area in the district around the workplace (see the first above reported excerpt), but also the introduction of vending machines with healthy snacks, and ‘recharge’ rooms for workers, on the model of big IT companies (e.g., Google). This demonstrated the willingness of the workers in participating to the design of the program (explicitly reported in the second excerpt), besides their health condition, suggesting services addressing the whole workforce. In the focus groups emerged the representation of the WHP program as yet another initiative of occupational welfare of the research center; as such, their expectation was of being aligned to the other services offered to all employees.

*Worker 3: when a Super-boss comes and says: “we thought of you!” I remain a little bit puzzled.*

*Worker 4: the official conference with the heads (of the center) in suits and making a boring speech (…) does not make sense! The project should be presented by someone who already does sports, such as someone from a recreational club. An expert, rather than a boss, could be important.*

*(Focus group 1 with the workers).*

Furthermore, the workers who participated in the focus group suggested how the project should be promoted to be attractive to the workforce. Sceptical of top-down interventions, focus group participants suggested peer-to-peer initiatives to be more effective. Following these suggestions, the project was launched with a kick-off meeting where, alongside the top management, researchers outlined the scientific basis of the program and emphasized the importance of a broader adhesion and participation. Beyond the possibility of enjoying the potential benefits offered by the program, participation in the program was also asked for supporting its evaluation and improvement.

*Worker 3: In my opinion, alcohol consumption is a touchy subject because the line between addiction and conscious consumption is very subtle. I may agree with eliminating alcoholic beverages from the canteen (…) but, from my perspective, promoting the total elimination of alcohol from the workers’ daily life is problematic (…). To intervene on drinking and smoking is fine when there is a form of addiction that interferes with the work. I do not think smoking 2–3 cigarettes per day is a form of addiction that impacts productivity and absenteeism (I guess not).*

*[Focus group 2 with the workers].*

The focus groups also uncovered the different meanings the workers attached to the four dimensions of health prevention. A clear distinction emerged between nutrition and physical activity on the one hand, and the consumption of alcohol and tobacco on the other hand. Workers appreciated the initiatives targeting the canteen as well as the supply of fitness courses offered at discounted rates. However, the participants to the focus group opposed measures addressing alcohol consumption and smoking, arguing that these habits refer to individual preferences. As underlined in the above reported excerpt, an intervention on alcohol consumption is tolerable only when this latter interferes on working activity, in terms of productivity or occupational safety.

Finally, workers expressed concerns regarding data protection, requiring that only the occupational physician should be given access to them and not share it with HR department. Although most workers recognized the fairness of HR in personal data management practices, they pointed out the need of balancing the trade-off between the epidemiologic purposes of the program and privacy concerns of their participants. They thus urged HR to assure the workers about privacy issues, and the policies of personal data protection.

The inclusion of workers in the focus groups contributed to change the design of the WHP initiative, translating it into a fully-fledged co-production process. As the WHP program crossed another social boundary, it was re-arranged to accommodate the visions and priorities of another actor in the process.

The final version of the project included most of the suggestions emerged from the focus groups. While the purpose of evaluating, preventing, and reducing health risks was maintained, several changes signaled the reconfiguration of the program:

- the risk assessment process, originally designed to identify the workers to be included in the program because of their risky health status, was redesigned also for providing a personalized evaluation of their health status from the occupational physician;
- all workers could download the mobile app, regardless of their health status (whereas this app was initially set up as a tool for workers with risky health conditions);
- with the help of the recreational club, some additional initiatives were developed: seminaries with nutritionists, courses of postural gym and a walking group;
- the program provided for an *in itinere* process of monitoring of the health conditions of participants: beyond tailoring and taking care of individual health conditions, this service was intended to gather information to improve the program and the activities it provided.

In the final version, the original focus of the program (the creation of a platform for helping workers with a high risk of developing cardiovascular diseases and/or Type 2 diabetes) became just one of the services (renamed as prevention services) within a broader program of workplace health promotion.

## Discussion

In this study we considered a WHP program as an arrangement that can foster the promotion of healthier behaviors by actors inhabiting intersecting social worlds, within and outside an organization. Starting from this assumption, we focused our analysis on the collaborative processes performed by the actors to re-shape the program.

From this viewpoint, we adopted the notions of boundary object and co-production as analytical tools that enable us to respond to two questions:

1. how do different actors come to agree on a shared workplace health promotion program?;
2. on what terms a workplace health promotion program become acceptable by its main intended beneficiaries, the employees?

### WHP program as a co-produced modular boundary object

The first question deals with the procedural aspects or the making of the WHP program and how consensus is built around it. The short answer to our question is that the final agreement was not the result of moving toward a shared definition of health prevention but rather the incorporation of the different perspectives of each stakeholder.

Our research shows how new stakeholders were identified and included at each step of the process. A main result of this study is that at each stage the WHP program is not altered but rather enriched. Each new stakeholder brings to the table their perspective and an updated version of the program addresses the concerns raised. As a result, the WHP initiative becomes a modular program which takes into account each and every issue raised by all the stakeholders at different stages of the process. Each actor inscribes its (his/her) perspectives in the WHP program which is not the result of a mediation rather the emerging product of the composition of each matter of concern.

For instance, the original program rooted in strong clinical perspective and addressed to employees with cardiovascular risk and aimed at a sub-set of the workforce of the research center is nor abandoned nor re-shaped. Rather, it is flanked by other initiatives designed to involve all employees excluded. Each specific initiative is the core concern of a sub-set of the stakeholders, and it is regarded with little interest by others.

Boundary objects, in their theoretical definition, highlight the conditions for collaborating even without consensus: agreements can be reached, after negotiations and compromises, although actors can fail in achieving them. In the case under analysis, the stakeholders find a common ground by adding new components to the original program. In this case, the co-production serves the purpose to allow partners to establish *“a shared language that can be used by different communities to present or represent their specific knowledge”,* allowing *“individuals from different communities to communicate across boundaries”* (Uppström and Lönn, 2017,409) while pursuing individual objectives.

The decision to ‘agreeing to disagree’ or take into account all the different perspectives emerged during the design of the WHP intervention is most likely motivated by the desire to avoid conflict. All the stakeholders involved in the project have a long-standing history of collaboration in several past and future projects which both favored the communication and led to circumvent all possible cases of disagreement by simply providing minimum or no collaboration to those parts of the protocol not directly of their interest. Besides its instrumentality in the making of the protocol, the co-production phases allowed the stakeholders to make clear which activities were relevant and what effort, if any, was to be expected from them in each of them.

### The boundaries of adherence

The second goal of our research was to investigate the willingness of employees to be enrolled in a health prevention program managed by their company. Employees’ participation to WHP programs is a significant issue since, despite all efforts of all the other stakeholders, the results will ultimately depend on workers voluntarily agree to espouse the initiative.

Our study explores willingness through the analysis of the representations of the WHP program emerging from the focus groups in which it was presented.

First and foremost, employees provided an alternative perspective to the WHP program, symbolically placed alongside the occupational welfare initiatives offered by the research center. Besides the intrinsic value of the health promotion, employees stressed the relevance of making the program available to all workers in analogy with services already available. In doing so, employees (unknowingly) strengthened the positions of the HR department and the recreational club among the stakeholders paving the way for some of the additional initiatives made available in the final program (e.g., postural gym classes for beginners).

Secondly, employees involved in the focus groups drew a line concerning what they deemed to be the legitimacy of employers’ intervention in the personal sphere of health promotion. With regard to the four pillars of prevention, employees regarded positively interventions addressing diet and physical activity while measures targeting smoking and alcohol consumption were described as intrusive. While employees did not deny the impact of smoking and drinking on health, these were represented as social activities concerning to the preferences of the individuals that should not be addressed by a company-led health initiative.

While employees did not take part to the meetings in which the details of the protocol were discussed, their inputs contributed to shape its final version.

The involvement of employees through the focus groups led to several changes of the final program, excluding specific measures targeting smoking and drinking. Moreover, the whole WHP program was rebranded as a part of the organizational welfare initiatives of the research center to leverage on the overall positive evaluation of such services.

With regard to the analytical lenses adopted in our study, our findings suggest that while employees were never formally involved in the co-production of the program, they were able to steer and shape the program by presenting the expected benefits and the limitation of a health prevention program before its implementation.

## Conclusion

WHP programs inhabit different social worlds, since they are prompted by supranational initiatives, national and regional policies, as well as by healthcare providers. Within this multi-layered institutional mobilization, each experience of promotion of workers’ well-being programs represents both an opportunity and a challenge for workers and companies, as they provide a new stake in the relation between these actors. From this point of view, our study sheds light on the issues that may emerge in the design and shaping of a WHP program during its development. This is a point scarcely discussed in the interdisciplinary literature on this topic.

Without having the improper ambition of generalizing the findings of our study, we propose some insights that may contribute to balancing the goals of WHP programs with the needs and preferences of their recipients, intended as actors who can have voice in its implementation rather than being mere recipients.

Firstly, our study has emphasized how the design of a WHP program can lead to disagreements between the stakeholders of the program. While this can seem highly expectable and obvious, we noticed that disagreements have been tackled by adopting a process of co-production of the program. One of the main implications of this process of co-production was the re-shaping of the boundaries of the program. Co-production supported the participation of a higher number of workers, questioning the top-down and asymmetric structure of the program. This occurred without reaching a full consensus between the actors, neither from its beginning nor in itinere. On this basis, we claim that co-production can be a pattern for designing WHP programs that match heterogenous expectations while fitting with their expected effectiveness (as stated by a plethora of empirical studies).

Secondly, we claim that WHP programs should be designed considering ethical concerns too, beyond medical protocols. These concerns relate to the possibility of refusing to participate in the programs by their recipients. From this point of view, the issue is not the compulsoriness of participation since workers participate voluntarily. Ethical concerns should deal with the intrusiveness of a WHP program on individual lifestyles and preferences. The improvement of a specific dimension of health status may be contested and rejected by some workers. While co-production can be a way to tackle this issue, we claim that major attention should be given to ethical concerns in the institutional promotion of WHP programs. This reasserts the necessity of properly marking the boundaries of a welcome yet challenging initiative.
